# Enhancing the growth performance of replacement female breeder goats through modification of feeding program

**DOI:** 10.14202/vetworld.2017.630-635

**Published:** 2017-06-11

**Authors:** A. A. A. Ghani, M. S. Shahudin, M. Zamri-Saad, A. B. Zuki, H. Wahid, A. Kasim, M. S. Salisi, Hasliza Abu Hassim

**Affiliations:** 1 Department of Veterinary Preclinical Sciences, Faculty of Veterinary Medicine, Universiti Putra Malaysia, 43400 Serdang, Selangor Darul Ehsan, Malaysia; 2 Research Centre for Ruminant Diseases, Faculty of Veterinary Medicine, Universiti Putra Malaysia, 43400 Serdang, Selangor Darul Ehsan, Malaysia; 3 Department of Animal Science, Faculty of Agriculture, Universiti Putra Malaysia, 43400 Serdang, Selangor Darul Ehsan, Malaysia

**Keywords:** feeding, goat, growth performance, replacement breeder

## Abstract

**Aim:**

The study was conducted at a smallholder goat farm located in Labu, Negeri Sembilan, Malaysia. The objective of this study was to evaluate the effect of proper feeding program on growth performances of replacement breeder goats.

**Materials and Methods:**

A total of 30 healthy female boer cross goats at the age of 4 months old with average initial live body weight (BW) of 20.05±0.5 kg were used for on-farm feeding trial to evaluate the growth performance as preparation for breeding purposes. The experimental goats were divided into two groups of 15 animals each labeled as control and treatment groups, which were kept under intensive farming system. Goats in control group were fed with normal routine feeding protocol practiced by the farmer, while goats in the treatment group were fed with new feed formulation. Throughout the experimental period, on-farm monitoring and data collection were carried out. Initial BW and body condition score (BCS) were recorded before the start of the experiment while final BW and BCS were gained after 7 months of the experimental period. Average daily gain (ADG) was calculated after the experiment end. Data on BW, ADG, and BCS were recorded from both groups for every 2 weeks and reported monthly. The feed intake for the control group was 2.8 kg/animal/day which practiced by the farmer and 3.2 kg/animal/day as new feed formulation for the treatment group.

**Results:**

After 7 months of the experimental period, final BW shows an improvement in treatment group (39.1±1.53 kg) compared with control group (32.3±1.23 kg). The ADG in treatment group also gives promising result when comparing with control group. Goats in treatment group significantly attained better ADG than control group which were 126.7 g/day and 83.3 g/day, respectively. For the BCS, goats in the treatment group had shown an improvement where 86.67% (13 out of 15) of the group had BCS ≥3 (1-5 scoring scale) and only 66.67% (10 out of 15) of the control group had BCS ≥3.

**Conclusion:**

Therefore, it was concluded that implementation of proper feeding program as shown in treatment group give promising result to improve the growth performance of replacement breeder goats which can be adopted by the farmers to improve farm productivity.

## Introduction

Postweaning phase, also known as grower phase, is a critical part where the ability of the farmers to manage this phase will determine the sustainability of the farm, regardless of the production system either for meat or milk. Within this phase, the kids are totally separated from the dam and able to consume solid feed as their rumen fully develop and were raised either as replacement herd or slaughter for meat. Postweaning period can be stressful and critical period resulting in weaning shock [1] and often affect the growth performance by slowing or stoppage of growth and even loss of weight gain [2].

In the aspect of nutritional management, adequate nutrient should be given to animal according to their sex, age, breed, production system (dairy or meat), body size, climate, and physiological stages [3]. It is important to ensure the animal diet is formulated to support optimal production and be economical [4]. For the selected kids to be replacement breeder that kept under intensive farming system, diets or feed must be carefully formulated to assure that all nutrient requirements of the animal are met as the animal have limited choice in selecting the feed. It is well known that deficit or excess of nutrients during growth has a negative effect on the production traits of female at maturity [5].

To be a potential replacement breeder especially female goat, it is important to have a good body condition and growth rate as well as proper weight for breeding. Unable to have these criteria may cause it to be rejected or culled from the replacement herd. The slow growth rate is mainly attributed to poor nutrition and management and nongenetic factors, such as season, sex, type of birth, and dam age or parity [6]. To majority of smallholder farmers, balance feeding is less concerned due to time, cost constrain, and labor issues. Indeed, imbalance feeding could affect the physiological function due to inadequate nutrient and further lead to reduction in growth performance and productivity of the farms. Unable to provide sufficient nutrients during postweaning may cause decreased kid growth, delay in puberty, lowered fertility, and lowered resistance to disease and parasites [7].

In Malaysia, most of the smallholder goat farms are not managed properly due to poor knowledge and information in standard management practice for goat farm, which further resulted in poor performance of these goat farms. Indeed, most of smallholder farm has never adopted any standard rearing management, particularly on feeding protocol which further resulted in lower performance of the goats with respect on growth and breeding performance, feed utilization and production. Thus, it is important to implement a proper feeding program to the replacement herd to ensure that the goats are ready and in good body condition for breeding as well as improvement in growth performance with respect to the body weight (BW), average daily gain (ADG), and body condition score (BCS). Therefore, the objective of this study is to evaluate the effect of implementation a proper feeding program on growth performances of replacement breeder goats as compared to the routine feeding program practice by the smallholder farmer under intensive farming system.

## Materials and Methods

### Ethical approval

The experimental protocol used in this study was approved by Institutional Animal Care and Use Committee (IACUC) with reference number UPM/IACUC/AUP-R039/2016, in accordance with the standard guidelines on usage and care of experimental animals.

### Experimental design

This study was conducted at a goat farm located in Labu, Negeri Sembilan, Malaysia. The total population of goat raised in this farm was considered small (n=100) as this farm was managed by a smallholder farmer. Thus, in this study, 30 healthy female boer cross goats at the age of 4 months old (postweaning) with average initial live BW of 20.05±0.5 kg were selected and used for on-farm feeding trial to evaluate the growth performance as preparation for breeding purposes. All goats were kept in the same housing area to reduce biases from environmental factors and were managed under intensive farming system. The goats were then divided equally into control (n=15) and treatment (n=15) groups. Each group was fed with different feed formulations, in which the control group was fed according to the routine feeding program by the farmer. Indeed, after a thorough screening and complete feed analysis was done, this feeding program was found inadequate and failed to meet the requirement in term of quantity and quality of the feed. As for the treatment group, the goats were fed with standard feeding program according to the recommendation by NRC [8]. In this study, both groups were fed with same local feed resources such as Napier grass, local plants which include *Macaranga* sp. and *Mallotus* sp., pressed soy waste and commercial concentrate with different proportions (Table-1). All goats were fed twice a day, which consists of pressed soy waste and commercial concentrate mixture in the morning (900 h) and Napier grass and local plants mixture in the evening (1700 h).

**Table-1:** Feed formulation for control and treatment groups.

Feed ingredients	Control group	Treatment group
Napier grass (kg)	1.2	1.2
*Mallotus* sp. (kg)	0.4	0.4
*Macaranga* sp. (kg)	0.4	0.4
Pressed soy waste (kg)	0.72	0.95
Commercial concentrate (kg)	0.08	0.25
Total feed given	2.8 kg animal/day	3.2 kg/animal/day

The feeding trial lasted for 7 months started from postweaning age (4 months old) until the goats reach the breeding criteria, approximately at the age of 11 months old. Throughout the experimental period, on-farm monitoring and data collection were carried out. Clean water was supplied *ad-libitum* during the feeding trial and anti-stress (Stress Pack^®^) was provided in drinking water every 2 weeks to reduce stress to the animals. Three parameters associated with growth performance were determined in this study which consists of BW, ADG, and BCS as described by Salisi [9].

### Feed analysis

Before the implementation of feeding trial was conducted, the nutrient composition (dry matter, crude protein, crude fiber, crude fat) for total mix ration of the feed given to the goats for each diet groups (control and treatment groups) were determined by proximate analysis. Proximate analysis is the most common analysis performed on feed samples, where it consists of a series of analyses to estimate the nutrient characteristic of feeds which includes the following: Dry matter, crude protein, crude fat, and crude fiber. All the analyses were performed according to certified procedures outlined by the Manual of Laboratory Techniques, Universiti Putra Malaysia which was developed according to procedures of AOAC [10]. The proximate analysis of the samples was performed in 4 replicates.

### Measurement of BW and ADG

Initial BWs of the experimental animals were taken at the beginning of the study by two consecutive weighing in the morning before feeding. Live weight gain of each animal was recorded at 14 days interval, at 800 h in the morning before feeding and reported monthly until at the end of 7 months of feeding trial period. ADG was calculated as the difference between final live weight and initial live weight divided by the number of days of the feeding trial. In this study, ADG was calculated for every 30 days.

### Measurement of BCS

BCS was measured using a scale of 1-5 as described by Koyuncu [11]. Determination of the scoring was made by several characteristics of the goats as stated in Table-2.

**Table-2:** Body condition score scale.

Scale	Descriptions
1 (very thin or emaciated)	Spinous processes are sharp and prominent. Transverse processes are sharp. No fat cover
2 (thin)	Spinous processes are less sharp and prominent. Transverse processes are sharp. Less fat cover
3 (medium or moderately lean)	Spinous processes are smooth and rounded. Transverse processes are smooth and well covered. Some fat cover. Ideal body condition score
4 (moderately fat)	Spinous and transverse processes well covered and rounded. Thick muscle
5 (very fat/obese)	Spinous processes cannot be detected. Muscle is very full with very thick fat cover

### Data analysis

Data from feeding trial (monthly BW, ADG, and BCS) for both groups were recorded for further analysis. Preliminary data analysis such as normality test and screening for outliers were performed before conducting the main data analysis. Data of BW and ADG from every month then were entered into SPSS software for t-test analysis at p<0.05 while the percentage of BCS were calculated using Microsoft Excel software.

## Results

### Proximate analysis

The result of proximate analysis for all feed formulation (control and treatment groups) was tabulated in Table-3.

**Table-3:** Nutrient composition of feed formulation in control and treatment groups.

Nutrient composition (%)	Mean±SEM	

	Control group	Treatment group
Dry matter	71.45±1.27^a^	72.58±0.82^a^
Crude protein	11.39±0.74^a^	16.04±0.61^b^
Crude fiber	20.44±0.35^a^	23.82±0.54^b^
Crude fat	2.21±0.24^a^	3.08±0.33^b^

a,b Different alphabets within a row indicate significant difference between means using t-test at p<0.05. SEM=Standard error of the mean

From Table-3, dry matter content in both feed formulation does not show a significant difference. As expected, crude protein, crude fiber, and crude fat content in treatment group were higher than control group and shown significant difference between both groups.

### BW

The first parameter to assess growth performance is BW. For the results of BW, at the beginning of experiment before feeding trial, the mean of initial BW in control and treatment groups almost similar which are 20.0±0.52 kg and 20.8±0.71 kg, respectively. Then, after 7 months of feeding trial, the mean of final BW in treatment group (39.1±1.53 kg) shown a significant improvement compared with control group (32.3±1.23 kg). BW gain in 7 months for control and treatment group was 12.3 kg and 18.3 kg, respectively. The monthly progressions were tabulated in Table-4 and expressed in kilogram.

**Table-4:** The measurement of body weight in control and treatment groups throughout feeding trial.

Age of animal	Duration of feeding trial	Mean±SEM		

		Control group (n=15)	Treatment group (n=15)
4 months old	Initial body weight	20.0±0.52^a^	20.8±0.71^a^
5 months old	1 month	21.1±0.48^a^	22.4±0.64^a^
6 months old	2 months	22.4±0.35^a^	24.2±0.51^a^
7 months old	3 months	23.9±0.54^a^	26.3±0.45^b^
8 months old	4 months	25.6±0.81^a^	28.8±0.87^b^
9 months old	5 months	27.6±1.13^a^	31.8±1.21^b^
10 months old	6 months	29.8±1.33^a^	35.3±1.18^b^
11 months old	7 months	32.3±1.23^a^	39.1±1.53^b^

a,b Different alphabets within a row indicate significant difference between means using t-test at p<0.05. SEM=Standard error of the mean

### ADG

The second parameter to assess growth performance is ADG. ADG is a reflection from the increment of the BW in a period of time. In this study, ADG was calculated every month for each animal until the end of the experimental period. After 7 month, ADG in treatment group give promising results compared with the control group. In the 1^st^ month of feeding trial, the mean of ADG for control and treatment groups were 36.7 g/day and 53.3 g/day, respectively. Then, after 7 month of feeding trial, the mean of ADG for control and treatment groups were 83.3 g/day and 126.7 g/day, respectively. The difference of ADG between control and treatment groups was increased from 16.6 g/day in the 1^st^ month to 43.4 g/day in the 7^th^ month of feeding trial. The monthly results of ADG were summarized in Table-5 and expressed in g/day.

**Table-5:** The measurement of average daily gain in control and treatment groups throughout feeding trial.

Age of animal	Duration of feeding trial	Mean±SEM	

		Control group (n=15)	Treatment group (n=15)
5 months old	1 month	36.7±2.11^a^	53.3±3.25^b^
6 months old	2 months	43.3±2.53^a^	60.0±3.46^b^
7 months old	3 months	50.0±3.22^a^	70.0±3.73^b^
8 months old	4 months	56.7±3.51^a^	83.3±4.13^b^
9 months old	5 months	66.7±2.98^a^	100.0±4.55^b^
10 months old	6 months	73.3±3.34^a^	116.7±4.28^b^
11 months old	7 months	83.3±4.07^a^	126.7±5.16^b^

a,b Different alphabets within a row indicate significant difference between means using t-test at p<0.05. SEM=Standard error of the mean

### BCS

The third parameter to assess growth performance is BCS. BCS system uses a scale from 1 (very thin) to 5 (very fat). At the beginning of this study, both groups have an equal percentage of animals that having BCS ≥3 which is 40.0% while the remaining having BCS <3. After 7 months of feeding trial, the percentage of BCS ≥3 in control and treatment groups was 60.0% and 86.7%, respectively. The monthly progressions were tabulated in Table-6.

**Table-6:** The measurement of body condition score in control and treatment groups throughout feeding trial.

Duration of feeding trial	Control group (n=15)		Treatment group (n=15)	
	
	BCS<3 (%)	BCS≥3 (%)	BCS<3 (%)	BCS≥3 (%)
Beginning	9 (60.0)	6 (40.0)	9 (60)	6 (40)
1 month	8 (53.3)	7 (46.7)	7 (46.7)	8 (53.3)
2 months	8 (53.3)	7 (46.7)	6 (40.0)	9 (60.0)
3 months	7 (46.7)	8 (53.3)	5 (33.3)	10 (66.7)
4 months	7 (46.7)	8 (53.3)	3 (20.0)	12 (80.0)
5 months	6 (40.0)	9 (60.0)	3 (20.0)	12 (80.0)
6 months	6 (40.0)	9 (60.0)	2 (13.3)	13 (86.7)
7 months	6 (40.0)	9 (60.0)	2 (13.3)	13 (86.7)

BCS=Body condition score

## Discussions

During postweaning or grower stage, providing adequate nutrients through proper feeding program is very important especially to the replacement herd as preparation for breeding purpose. Nutrients composition of feedstuffs and feed intake by the animal should be considered during formulating the total mix ration. Within this stage, energy and protein are the greater nutrients need by the young goats to build new tissues for growth or replacement in an animal body [12]. In this study, the feed ingredients used are similar for both groups except the proportion of pressed soy waste and commercial concentrate which are different. Additional 230 g of pressed soy waste and 170 g of commercial concentrate were included in treatment group as protein and energy source, respectively. As animal grows from 6 months to breeding age, they may require at least 14-16% protein. Feeding more than 25% is not recommended for rapidly growing replacement animals [13].

On the aspect of BW, proper feeding program should be able to improve BW to gain desired BW for breeding. The proper time of breeding depends on the weight and age of the doe. Proper weight for breeding in does usually when the animal attained 60-70% of its mature BW. According to Grayling [14], recommended BW at first estrus for boer female goats is 31.3 kg. Thus, in this study, it revealed that goats from treatment group reach the minimum BW for breeding with weight of 31.8±1.21 kg at the age 9 months old while goats from control group reach the minimum BW for breeding with weight of 32.3±1.23 kg at the age 11 months old. It shown those goats in treatment group can be bred 2 months earlier than goats in control group. This supported by Jaudas and Mobini [15] where the study stated that a normally developed young doe can be bred at the age of about 9 months old without problem and will kid for the first time at the age of 14 months. Furthermore, feed formulation given to the goats in treatment group give promising improvement to BW gain compared with control group.

BCS is one of the growth performance parameters used in the evaluation and management of goat herd [16]. BCS can be defined as the level of fatness or thinness of the animal. BCS system uses a scale from 1 (very thin) to 5 (very fat). Within grower stage, monitoring on BCS very important to determine feed convertibility, health status, ability to gain, and establishing the condition of animal during routine management as well as related to fertility in breeding animals. As for breeding preparation, replacement doe should be raised under conditioning process which means to gain desired ideal body condition and BW. The recommended ideal BCS for breeding is 3-3.5 [17]. Goats that having poor BCS (<3) should receive flushing to reach ideal score while goats are too fat (BCS more than 4) should be fed with low energy feed to reduce BCS to ideal range. In addition, does in greater BCS having high ovulation rate which will increase the chances to get pregnant compared with does in poor BCS [18,19]. Replacement goats that are supplemented with high energy and protein before breeding have greater body condition subsequently leads to normal estrous cycles and increase the ovulation rate. Female goats with poor body condition have abnormal estrous cycles and fewer ovulations than female goats in greater body condition [20]. In this study, it revealed goats in treatment group that had BCS ≥3 increased significantly from 40.0% at the beginning to 86.7% after 7 months of feeding trial and ready to be bred support by meeting the desired BW for breeding. Meanwhile, in control group, only 60.0% of the goats had BCS ≥3 after 7 months of feeding trial and ready to be bred. BCS also can be used to evaluate the health status of the animals. For example, sick goats that suffering from gastrointestinal parasitism had poor BCS with other clinical signs such as scouring, unthriftiness, weight lost, reduce weight gain, and reproductive inefficiency [21].

## Conclusion

After 7 months of feeding trial, all parameters for assessing the growth performance of animals in treatment group had shown the promising result when compared with control group. It is extremely important to meet the goat’s requirement by providing and implementing a proper feeding program to improve the performance of replacement goats, especially growth performance as well as to increase the productivity of the farm.

## Authors’ Contributions

All authors were involved and contributed in this research works. AAAG, HAH, MSS, MZS, ABZ, HW, AK and MSS were involved in experimental trial and data collection. AAAG and HAH wrote and edited the manuscript according to the title. All authors read and approved the final manuscript.
